# A combinatory ferroelectric compound bridging simple ABO_3_ and A-site-ordered quadruple perovskite

**DOI:** 10.1038/s41467-020-20833-6

**Published:** 2021-02-02

**Authors:** Jianfa Zhao, Jiacheng Gao, Wenmin Li, Yuting Qian, Xudong Shen, Xiao Wang, Xi Shen, Zhiwei Hu, Cheng Dong, Qingzhen Huang, Lipeng Cao, Zhi Li, Jun Zhang, Chongwen Ren, Lei Duan, Qingqing Liu, Richeng Yu, Yang Ren, Shih-Chang Weng, Hong-Ji Lin, Chien-Te Chen, Liu-Hao Tjeng, Youwen Long, Zheng Deng, Jinlong Zhu, Xiancheng Wang, Hongming Weng, Runze Yu, Martha Greenblatt, Changqing Jin

**Affiliations:** 1 Beijing National Laboratory for Condensed Matter Physics, Institute of Physics, Chinese Academy of Sciences; School of Physical Sciences, University of Chinese Academy of Sciences, Beijing, 100190 China; 2 Songshan Lake Materials Laboratory, Dongguan, 523808 China; 3 Max Planck Institute for Chemical Physics of Solids, Nothnitzer Straße 40, Dresden, 01187 Germany; 4 School of Advanced Materials, Peking University Shenzhen Graduate School, Shenzhen, 518055 China; 5 NIST Center for Neutron Research, National Institute of Standards and Technology, Gaithersburg, 20899 MA USA; 6 School of Materials Science and Engineering, Nanjing University of Science and Technology, Nanjing, 210094 China; 7 X-ray Science Division, Advanced Photon Source, Argonne National Laboratory, Argonne, 60439 IL USA; 8 National Synchrotron Radiation Research Center, 101 Hsin-Ann Road, Hsinchu, 30076 Taiwan; 9 Department of Physics, Southern University of Science and Technology, Shenzhen, 518055 China; 10 Department of Chemistry and Chemical Biology, Rutgers, The State University of New Jersey, Piscataway, NJ USA

**Keywords:** Solid-state chemistry, Electronic properties and materials

## Abstract

The simple ABO_3_ and A-site-ordered AA′_3_B_4_O_12_ perovskites represent two types of classical perovskite functional materials. There are well-known simple perovskites with ferroelectric properties, while there is still no report of ferroelectricity due to symmetry breaking transition in A-site-ordered quadruple perovskites. Here we report the high pressure synthesis of an A-site-ordered perovskite PbHg_3_Ti_4_O_12_, the only known quadruple perovskite that transforms from high-temperature centrosymmetric paraelectric phase to low-temperature non-centrosymmetric ferroelectric phase. The coordination chemistry of Hg^2+^ is changed from square planar as in typical A-site-ordered quadruple perovskite to a rare stereo type with 8 ligands in PbHg_3_Ti_4_O_12_. Thus PbHg_3_Ti_4_O_12_ appears to be a combinatory link from simple ABO_3_ perovskites to A-site-ordered AA′_3_Ti_4_O_12_ perovskites, sharing both displacive ferroelectricity with former and structure coordination with latter. This is the only example so far showing ferroelectricity due to symmetry breaking phase transition in AA′_3_B_4_O_12_-type A-site-ordered perovskites, and opens a direction to search for ferroelectric materials.

## Abstract (web summary)

There are few reports of ferroelectricity due to symmetry breaking transition in A-site-ordered quadruple perovskites. Here, the authors find one with phase transition from a high-temperature centrosymmetric paraelectric phase to a low-temperature non-centrosymmetric ferroelectric phase in a high pressure synthesized compound.

## Introduction

Perovskites and their derivatives show many interesting physical, chemical and mineral properties such as ferromagnetism, ferroelectricity, piezoelectricity, ion conductivity, photocatalysis and superconductivity^1–18^ that can be modified dramatically by the coordination chemistry for a given composition. There is a special class of perovskite-type materials with the general chemical formula AA′_3_B_4_O_12_, named A-site-ordered, or quadruple perovskites, which received much attention owing to their fascinating structural and wide varieties of physical properties^10^, including colossal magnetoresistance under weak field^11^, charge disproportionation^12^ and giant dielectric constant over a wide temperature range^13,14^. For the simple ABO_3_ perovskite, the 12-fold coordinated A-site is often occupied by large size ions such as alkali metal, alkaline earth or lanthanide cations and the 6-fold coordinated B-site is often occupied by transition metals (TMs) to satisfy the so called tolerance factor *t* = (r_A_ + r_O_)/√2(r_B_ + r_O_) (r_A_, r_B_, & r_O_ represent for the ion radius of A, B, and O, respectively) with *t* usually in the range of 0.75 to 1.05 for stable perovskite compounds^1,18^. However in the A-site-ordered perovskite AA′_3_B_4_O_12_, three quarters of the A-site is substituted by a TM, A′ with much smaller ionic radius^16–19^. Generally, TM ions with strong Jahn-Teller distortions like Mn^3+^ and Cu^2+^ preferentially occupy the A′-site with square-planar coordination^20–24^. The small TM ion at the A′-site causes the distortion of BO_6_ octahedron in AA′_3_B_4_O_12_ perovskite, usually resulting in a cubic crystal structure with space group *Im*-3^25^. From a structural point of view, the flexibility for tilting the BO_6_ octahedra in AA′_3_B_4_O_12_ perovskite is limited in order to maintain the square-planar coordination at the originally 12-fold coordinated A-site in the simple perovskite structure^21^.

Ferroelectricity is one of the most important properties for application in actuators, sensors, and memory storage devices, etc. Large numbers of ferroelectric materials possess simple perovskite structures, such as BaTiO_3_, PbTiO_3_, Pb(Ti, Zr)O_3_, BiFeO_3_^26–30^. The origin of ferroelectricity is due to either the lone pair 6*s*^2^ effect of cations (Pb^2+^, Bi^3+^), or the second-order Jahn-Teller active cations with electron configuration of *d*^0^ (Ti^4+^, Zr^4+^); these compounds are usually referred to as displacive-type ferroelectrics. However, ferroelectricity is seldom observed in AA′_3_B_4_O_12_ type A-site-ordered compounds, even when they contain second-order Jahn-Teller active cations or lone pair effect cations^28^. One reason for the absence of ferroelectricity in this series of compounds is the usual presence of a centrosymmetric structure, and the variation of temperature generally causes isostructural or centrosymmetric - to - centrosymmetric phase transition^12,21,25^, hence there cannot be spontaneous polarization. Currently the rare examples in this series that show ferroelectricity are AMn_7_O_12_ (A = Ca, Bi or Pb)^31–33^ and AMn_3_Cr_4_O_12_ (A = La or Bi)^20,22^. Although these compounds have centrosymmetric structures, all are spin-driven multiferroic systems, due to strong magnetoelectric coupling effects^20,22,32^. Up to now, there is still no report of ferroelectricity from symmetry breaking transition in A-site-ordered perovskite compounds.

In this work, an AA′_3_B_4_O_12_ type A-site-ordered perovskite oxide, PbHg_3_Ti_4_O_12_ (henceforth PHTO) was designed and synthesized at high pressure and high temperature conditions. It is found that the Hg^2+^ ion occupies the A′-site in AA′_3_B_4_O_12_ type perovskite in sharp contrast to previous reports where 3*d* TM ions (Cu^2+^, Mn^3+^) usually locate at the A′-site. Moreover, ferroelectricity was observed in PHTO, when its ambient centrosymmetric phase transformed to a non-centrosymmetric phase at 250 K. The origins of ferroelectricity are discussed based on the comprehensive characterizations of crystal structure using both neutron and synchrotron x-ray diffractions and density functional theory (DFT) calculation.

## Results and Discussion

### Crystal structure

**Fig. 1:**
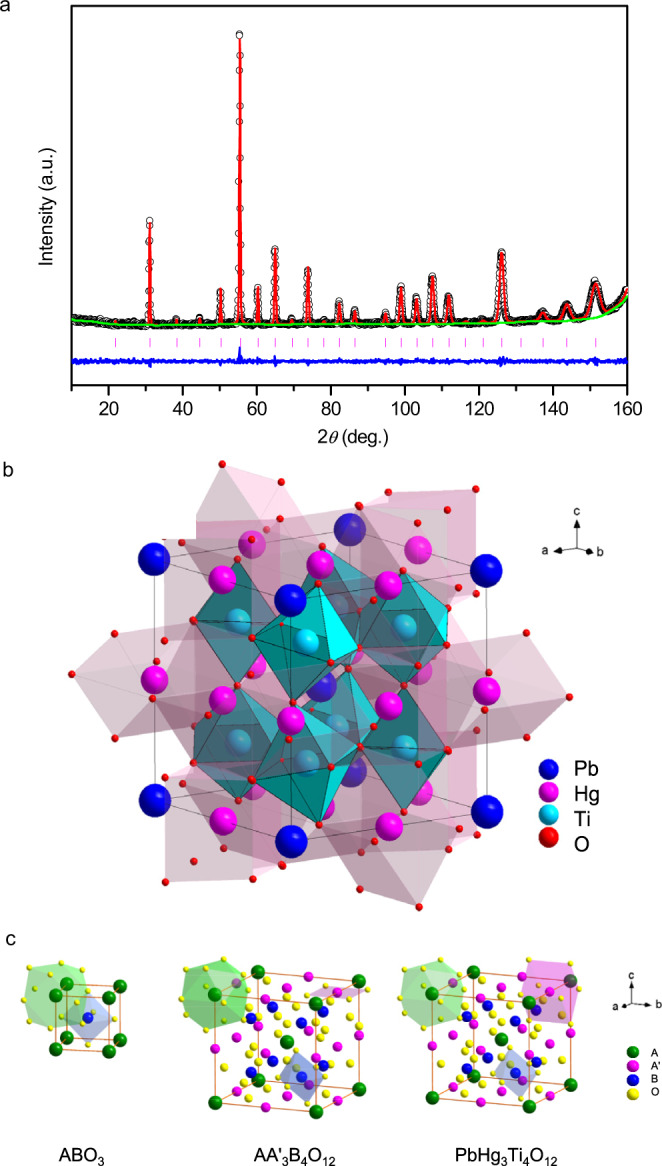
Neutron diffraction and crystal structure of PHTO. **a** Rietveld refinements based on NPD data at 295 K. Observed (crosses), calculated (red), difference (blue) are shown in the figure, respectively. The ticks indicate the allowed Bragg reflections with space group *Im*-3. **b** Crystal structure of PHTO. **c**, Schematic coordination of A, A′ and B-site for ABO_3_, AA′_3_B_4_O_12_ and PbHg_3_Ti_4_O_12_ respectively.

The NPD refinements are shown in Fig. 1a, which confirm that PHTO crystallizes in the AA′_3_B_4_O_12_ type A-site-ordered perovskite. The refined structure parameters of PHTO based on NPD data collected at 295 K are listed in Table 1. No anomaly is observed in the occupation parameters at any site, including full occupancy for the oxygen site as well. Thus, PHTO should have stoichiometric composition. Bond valence sum (BVS) calculations based on the refined structure from NPD data give valences of +2.26 for Pb ions, +1.99 for Hg ions and +3.88 for Ti ions (see Table 1), which are consistent with x-ray absorption spectroscopy (XAS) results discussed later. Hence the obtained sample is an A-site-ordered perovskite with stoichiometric Pb^2+^Hg^2+^_3_Ti^4+^_4_O_12_ formula and space group *Im*-3 as shown in Fig. 1b. The structure model is consistent with the refinements of SXRD data as shown in Supplementary Fig. S1 and Table S1. Moreover, for Hg ions, if one considers the four nearest - neighbor O atoms (Hg-O(×4) = 2.320(5) Å), the BVS value is only 1.56. However, if one takes into accounts the four next - nearest - neighbor O atoms (Hg-O(×4) = 2.798(1) Å) additionally, the BVS value is 1.99. Therefore, Hg ions at A′-site are close to 8 -coordinated by O, in sharp contrast to typical A-site-ordered perovskite where the A′-site is usually 4-coordinated by O (See Fig. 1c). Note that, the PHTO sample is single phase of high quality, no detectable diffraction peak belonging to PbTiO_3_ or HgTiO_3_ was found in either NPD or SXRD patterns.

**Table 1:** Refined structure parameters of PHTO based on NPD data collected at 295 K.

	NPD
a(Å)	7.7283(2)
Z	2
Formula weight (g/mol)	1192.56
Cacl. Density (g/cm^3^)	8.5800(2)
V(Å^3^)	461.57(9)
O_y_	0.7155(1)
O_z_	0.2090(7)
*U*_iso_(Pb)(Å^2^)	0.009(5)
*U*_iso_(Hg)(Å^2^)	0.014(2)
*U*_iso_(Ti)(Å^2^)	0.010(0)
*U*_iso_(O)(Å^2^)	0.014(0)
Pb-O(×12)(Å)	2.728(5)
Hg-O(×4)(Å)	2.320(5)
Hg-O(×4)(Å)	2.798(1)
Ti-O(×6)(Å)	1.975(8)
∠Ti-O-Ti (°)	155.83(6)
BVS(Pb)	2.26
BVS(Hg)	1.99
BVS(Ti)	3.88
R_wp_(%)	4.56
R_p_(%)	4.12

The BVS values (*V*_*i*_) were calculated using the formula *V*_*i*_ = Σ_*j*_*S*_*ij*_, and *S*_*ij*_ = exp[(*r*_0_ − *r*_*ij*_)/0.37)]. In PHTO, *r*_0_ = 2.112 for Pb, 1.972 for Hg and 1.815 for Ti. For the A-site Pb, 12-coordinated oxygen atoms were used. For the A′-site Hg, 8-coordinated oxygen atoms were used. For the B-site Ti, 6-coordinated oxygen atoms were used. ^b^Space group: *Im*-3; Atomic sites: Pb 2*a* (0, 0, 0), Hg 6*b* (0, 0.5, 0.5), Ti 8*c* (0.25, 0.25, 0.25), O 24 *g* (*0*, *y*, z).

**Fig. 2:**
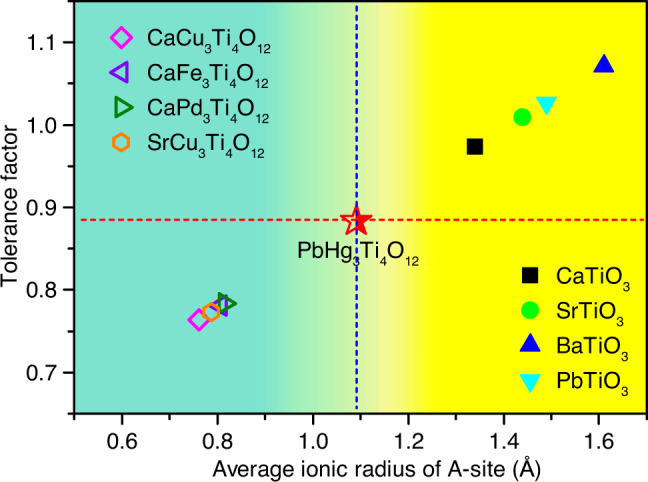
Tolerance factor *t* versus average ionic radius of A-site for simple perovskites ATiO_3_ and A-site-ordered perovskites AA′_3_Ti_4_O_12_. Upper right panel is the area for simple ATiO_3_ perovskites; Lower left panel is the area for AA′_3_Ti_4_O_12_ perovskites; The middle point is PHTO.

As aforementioned, usually the strong Jahn-Teller active ions, like Cu^2+^ and Mn^3+^, are preferentially accommodated into the square-coordinated A′-site in the A-site-ordered perovskites, as in LaCu_3_Fe_4_O_12_ and BiMn_3_Cr_4_O_12_^20,21^. Some other TM ions have been recently introduced at the A′-site such as in CaCo_3_V_4_O_12_^34^, CaFe_3_Ti_4_O_12_^35^, CaPd_3_Ti_4_O_12_^36^. Thus PHTO is an AA′_3_B_4_O_12_ type perovskites with the A′-site fully occupied by Hg^2+^, other than 3*d* TM, or Pd as shown in Table 2. The lattice parameter (7.72 Å) of PHTO is larger compared to those of other AA′_3_B_4_O_12_ type perovskites (7.3–7.5 Å), while the ∠Ti–O–Ti angle (155.8°) of PHTO is less distorted from the ideal 180° than those for other AA′_3_B_4_O_12_ type perovskites (∠*B*–*O*–*B* ≈ 140°). The larger ∠Ti–O–Ti angle and correspondingly smaller ∠A′−O−Ti angle in PHTO imply the less tilting of TiO_6_ octahedron, which is attributed to the large ionic size of Hg^2+^ about 0.96 Å. Figure 2 shows the tolerance factor *t* versus average ionic radius of A-site ions for simple perovskites ATiO_3_ and A-site-ordered perovskites AA′_3_Ti_4_O_12_ where the *t* factor is calculated assuming a simple ABO_3_ form with the average ionic size at A site. It is found that from CaTiO_3_ to BaTiO_3_, the ionic radius of A-site increases from 1.34 Å to 1.61 Å while the tolerance factor *t* increases from 0.97 to 1.07. The simple perovskites ATiO_3_ are located at the upper right panel of Fig. 2. For most of the AA′_3_Ti_4_O_12_ compounds, the average ionic radius of A-site is about 0.8 Å and the tolerance factor *t* is around 0.77. Therefore, AA′_3_Ti_4_O_12_ perovskites are located at the lower left panel of the Fig. 2. However, different from these ATiO_3_ and AA′_3_Ti_4_O_12_ compounds, the average ionic radius of A-site ion for PHTO is large, about 1.1 Å and the tolerance factor *t* is about 0.88. Moreover as aforementioned, the coordination number for Hg ions at the A′-site in PHTO is eight, not square planar as in A′-TM quadruple perovskites AA′_3_Ti_4_O_12_, and it is located in the middle area of Fig. 2, between those of simple ATiO_3_ (coordination number 12) and AA′_3_Ti_4_O_12_ quadruple perovskites (coordination number 4). Thus, PHTO appears to be a link from simple ATiO_3_ perovskite to AA′_3_Ti_4_O_12_ type A-site-ordered perovskite (Fig. 2). The A-site coordination number for ABO_3_ perovskite is 12 while the A-site and A’-site coordination numbers in the A-site-ordered quadruple perovskite AA'_3_B_4_O_12_ compound are 12 and 4 respectively. However in PHTO, the coordination number of A′ (Hg^2+^) is 8, which is just in between that for simple ATiO_3_ and AA'_3_Ti_4_O_12._ This result provides further evidence supporting the scenario aforementioned. Considering that the ionic size of Sr^2+^ (1.45 Å) is similar to that Pb^2+^ (1.49 Å), we also fabricated the A-site-ordered perovskite compound SrHg_3_Ti_4_O_12_, which is isostructural with PHTO, but without the observation of ferroelectricity. The result about SrHg_3_Ti_4_O_12_ will be published elsewhere. According to  the previous report^37^, the low energy level of *d*^10^ orbital prefers a relatively high coordination number owing to the weak *d*-*s* and *d*-*p* hybridization in perovskite compounds. As to PHTO system, the first principles calculation result indicates that the energy level of *d*^10^ for Hg^2+^ has a lower energy for 8 coordination than that for 4 coordination (See Supplementary Fig. S2a). Moreover PHTO also shows a low total energy in the state of 8 coordination of Hg^2+^ (See Supplementary Fig. S2b). Thus 8-fold coordination of Hg^2+^ is more stable than 4-fold in the PHTO system.

**Table 2:** Lattice constant *a*, ionic radius of A-site *r*_A_, ionic radius of A′-site *r*_A′_, difference between ionic radii of A- and A′-site ions Δ*r*_A_, metal−oxygen bond angles ∠A′−O−Ti and ∠Ti−O−Ti for some AA′_3_Ti_4_O_12_ type perovskite compounds^33^.

Compounds	*a*/Å	*r*_A_/Å	*r*_A_/Å	Δ*r*_A_/Å	∠A′−O−Ti/deg	∠Ti−O−Ti/deg
**PbHg**_**3**_**Ti**_**4**_**O**_**12**_	7.7234	1.49	0.96	0.53	102.60	154.22
**CaPd**_**3**_**Ti**_**4**_**O**_**12**_	7.4977	1.34	0.64	0.70	107.17	144.93
**CaFe**_**3**_**Ti**_**4**_**O**_**12**_	7.4672	1.34	0.64	0.70	107.82	144.01
**CaCu**_**3**_**Ti**_**4**_**O**_**12**_	7.3730	1.34	0.57	0.77	108.98	141.33
**SrCu**_**3**_**Ti**_**4**_**O**_**12**_	7.4275	1.44	0.57	0.87	109.18	141.21

### Valence state analysis

**Fig. 3:**
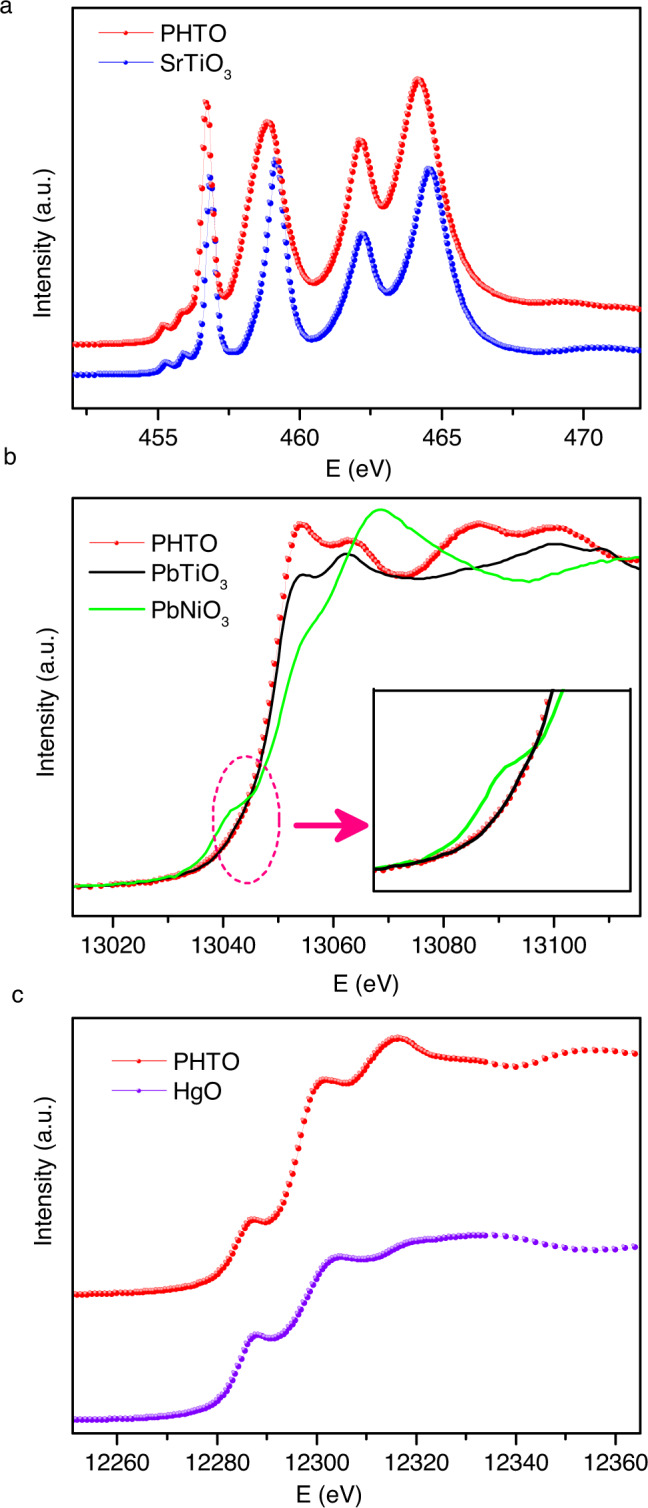
X-ray absorption spectroscopy of PHTO. **a** Ti-*L*_2,3_ edge. **b** Pb-*L*_*3*_ edge. **c** Hg-*L*_*3*_ edge.

Soft X-ray absorption spectroscopy at the *L*_2,3_ edge of TM *3d* ions^38^ is a highly sensitive method to determine the valence state^39,40^, local environment^41^, and orbital occupation^42^ of the ions. Figure 3a shows the Ti-*L*_2,3_ XAS spectra of PHTO together with SrTiO_3_ as a pure Ti^4+^ reference. The very similar multiplet spectral features in PHTO and SrTiO_3_ clearly correspond to the Ti 2*p*^6^3*d*^0^ → 2*p*^5^3*d*^1^ transition^43,44^, demonstrating the Ti^4+^ valence state in PHTO. Note that the PHTO spectrum is shifted by about 0.25 eV to lower energy with respect to the SrTiO_3_ spectrum, reflecting the different Ti local environments. Actually the Ti-O distance is 1.974(7) Å in PHTO and 1.951(7) Å in SrTiO_3_^45^, which suggests a weaker crystal field effect in PHTO. Figure 3b shows the Pb-*L*_3_ XAS spectra of PHTO together with PbTiO_3_ as a Pb^2+^ and PbNiO_3_ as Pb^4+^ reference. The weak pre-edge feature in PbNiO_3_ due to excitation from a 2*p* core electron to the 6*s* orbital. It has been established that this weak pre-edge feature is a sensitive finger-print of the Pb valence state in solid state materials^46^. The reason is that the Pb 5*d*^10^ orbitals are fully occupied, the valence state is then reflected by the 6*s* occupation. However, as shown in the inset, PHTO has the Pb-*L*_3_ XAS profile similar to the Pb^2+^ reference PbTiO_3_ in detail, no 2*p*–6*s* related excitation is observed in PHTO, which indicates the valence state of Pb^2+^ with fully occupied 6*s*^2^ state in this compound. Figure 3c shows the Hg-*L*_3_ XAS spectra of PHTO as well as HgO. The same energy position and very similar spectral profile of both PHTO and HgO indicate the Hg^2+^ valence state. The XAS results show that the electronic configuration of PHTO is Pb^2+^Hg^2+^_3_Ti^4+^_4_O_12_, which is fully consistent with the BVS results based on the crystal structure.

### Magnetic and transport characterizations

The magnetic susceptibility *χ* is almost constant and negative near zero from 300 K to 30 K, while *χ* increases rapidly on further decrease of temperature as shown in Supplementary Fig. S3. These results revealed that PHTO is nonmagnetic due to the 3*d*^0^, 5*d*^10^ and 6*s*^2^ electron configuration for Ti^4+^, Hg^2+^ and Pb^2+^, respectively, and consistent with the BVS calculations and XAS results. The resistance of PHTO at room temperature is too large to be measured, indicating the high electrical insulating property and few vacancies in the sample. Supplementary Fig. S4a shows the Ultraviolet-Visible-Near Infrared (UV-Vis-NIR) absorption spectrum of PHTO. The curve exhibits a steep decrease from 420 to 640 nm, which is a typical optical response of semiconductors. The optical band gap of the PHTO was estimated to be 2.12 eV. The first principles calculations show that PHTO is a direct gap insulator with 1.70 eV gap at the *H* point shown in Supplementary Fig. S4b. The gap might be underestimated in DFT calculations, nevertheless, it reveals the wide gap nature of PHTO. The obtained PHTO sample is stable at ambient up to 973 K as revealed by thermogravimetry analysis measurements shown in Supplementary Fig. S5.

### Dielectricity and ferroelectricity

**Fig. 4:**
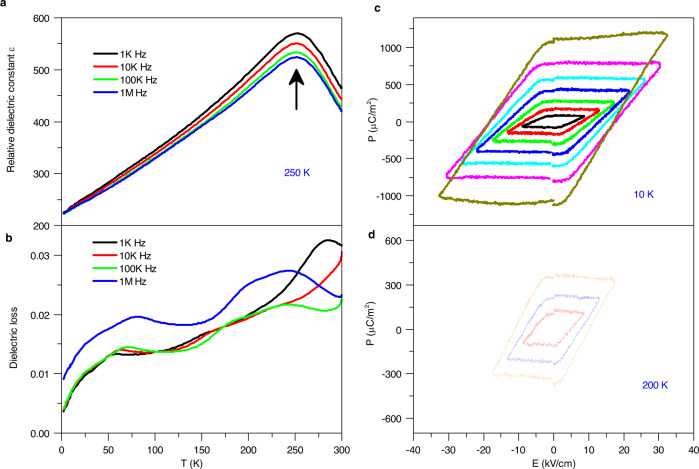
Dielectricity and ferroelectricity of PHTO. Temperature dependence of **a** relative dielectric constant *ε*_*r*_, and **b** dielectric loss at selected frequencies. The *P–E* hysteresis loops measured at **c** 10 K and **d** 200 K under selected electric fields.

The permittivity and dielectric loss measured with different frequencies from 2 K to 300 K are shown in Fig. 4a, b, respectively. The relative dielectric constant, *ε*_*r*_ of PHTO is over 220 in the measured temperature range. The temperature dependence of *ε*_r_ shows a clear peak around 250 K at all the frequencies of measurements. The overall temperature-dependent *ε*_*r*_ strongly indicates a low-temperature ferroelectric phase transition to a high-temperature paraelectric phase at *T*_FE_ ≈ 250 K. Furthermore, the transition temperature is independent of measurement frequency, indicating that PHTO is not a relaxor-type ferroelectric material. The relative increase of the dielectric loss around 250 K is consistent with the paraelectric-ferroelectric transition. Moreover the dielectric loss keeps at a very low value (<0.03) with all of frequency in the whole measurement range, which indicate the intrinsic dielectric property of PHTO. In order to further identify the ferroelectricity of PHTO, the isothermal polarizations were measured by the PUND method^20^. No loops of polarization versus electric field (*P*–*E*) were observed in polycrystalline PHTO at room temperature. However, canonical *P*–*E* hysteresis loops were observed below 250 K as shown in Fig. 4c, d. It is clear that polarization is enhanced at low temperature. At fixed temperature, the *P*–*E* loops significantly expand with increasing electric field. For example, the *P* value increases from 0.028 to 0.11 µC cm^−2^ as the maximum electric field changes from 17 to 32 kV cm^−1^ at 10 K. As known, PbTiO_3_ is a classic and extensively studied ferroelectric material with Curie temperature about 763 K, which excludes the possibility that the ferroelectricity of PHTO originated from PbTiO_3_. Thus it can be concluded that the ferroelectricity is due to PHTO.

### Temperature dependent crystal structure transition

**Fig. 5:**
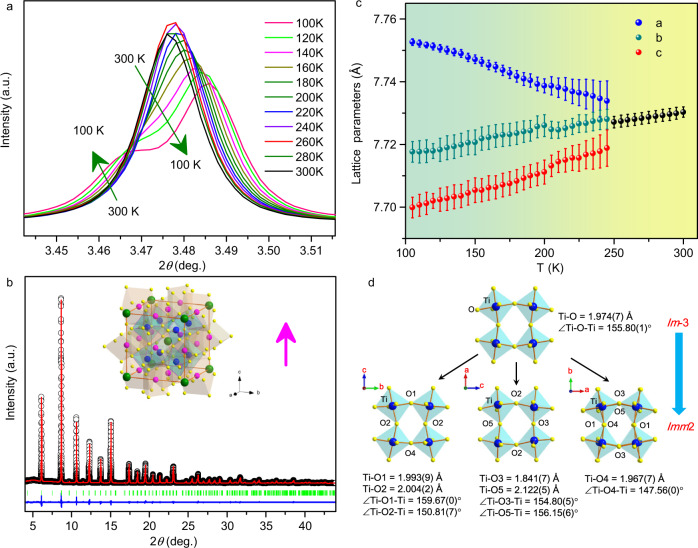
Temperature induced structural transition in PHTO. **a** The characteristic diffraction peaks (400) collected at different temperatures. **b** Rietveld refinements based on SXRD data at 90 K. The arrow denotes the polarization direction. **c** Temperature dependence of the lattice constant and **d** schematic illustration for the changing of TiO_6_ octahedron along different axis.

To further understand the mechanism of ferroelectricity from the structure point of view, we performed the temperature dependent SXRD, as shown in Supplementary Fig. S6. The characteristic (400) diffraction peak at different temperatures is collected in Fig. 5a. It is obvious that the diffraction peaks split into two sub-peaks between 240 K and 260 K, which indicates the occurrence of temperature induced phase transition at 250 K is due to symmetry reduction from a cubic - to - orthorhombic phase. Checking the SXRD data measured at 300 K and 90 K, all the high symmetry diffraction peaks, such as (200), (220), (222), (400) and (402) of the high temperature phase, are split into two sub-peaks on cooling to low temperature (see Supplementary Fig. S7). There is a clear long-range structural transition with decreasing temperature. The SXRD data collected at 90 K is presented in Fig. 5b. The crystal structure of PHTO can be fitted very well by an orthorhombic phase of non-centrosymmetric space group *Imm*2 (No.44). The inset shows the crystal structure of the low temperature phase. The Rietveld refinement results are listed in Supplementary Table S2. Figure 5c shows variation of the lattice parameters based on refinements of SXRD data. The lattice parameter *a* decrease lineally from 300 K to 250 K. Below the temperature of the phase transition, the lattice parameter *b* and *c* continue to decrease while parameter *a* increases. Figure 5d shows the evolution of TiO_6_ octahedron before and after phase transition. In the high temperature phase, TiO_6_ forms a regular octahedron with ∠Ti–O–Ti angle of 155.8° and Ti-O bond length of 1.974 Å respectively. When this cubic phase transformed to an orthorhombic non-centrosymmetric phase below 250 K, both ∠Ti–O–Ti angles and Ti-O bond lengths divergences occurred in TiO_6_ octahedron. These distortions originate from the relative displacements of Ti and O around all the axis directions. The polarization in the *ab*-plane was canceled due to the random relative displacements of Ti and O, leaving the polarization vector only along the [001] direction. As to HgO_8_ polyhedron, the single type HgO_8_ polyhedron with only two-types of Hg-O bond length in the high temperature phase evolves into three different type of HgO_8_ polyhedron with different bond length in the low temperature phase, but without contribution to the polarization. Supplementary Fig. S8 illustrates the evolution of HgO_8_ during the paraelectric-ferroelectric transition. Therefore, PHTO is a displacive-type ferroelectric. The ionic spontaneous polarization can be calculated by multiplying the effective charges and the distance between the negative and positive valence weighted mean center along the *c*-axis then divided by the volume of the unit cell^47^. Based on the refined results of NPD data collected at 5 K in Table 3, the calculated polarization value is about 13.65 μC cm^−2^. Although temperature-dependent phase transition is typical in A-site-ordered perovskites, it is usually isostructural or centrosymmetric - to - centrosymmetric phase transition from *Im*-3 to *Pn*-3. Most of those phase transitions are mainly induced by charge transfer between the A′-site and B-site ions or charge disproportionation at B-site ions^12,21,25^. PHTO is an example of AA′_3_B_4_O_12_ type A-site-ordered perovskite that exhibits a centrosymmetric to non-centrosymmetric phase transition.

**Table 3:** Refined structure parameters of PHTO based on NPD data collected at 5 K.

Crystallographic data for PbHg_3_Ti_4_O_12_ based on NPD at 5 K					
Atom	Wyck	x	y	z	*U*_iso_ (Å^2^)
Pb	2a	0.5	0.5	0.5	0.008(8)
Hg1	2b	0.5	0	0.5	0.007(1)
Hg2	2a	0.5	0.5	0	0.005(1)
Hg3	2b	0.5	0	0	0.005(4)
Ti	8e	0.2353(7)	0.2396(3)	0.2455(8)	0.013(9)
O1	4c	0.1966(0)	0	0.2905(6)	0.007(0)
O2	8e	0.2786(9)	0.2103(7)	0.0006(9)	0.013(4)
O3	4d	0	0.2802(5)	0.2527(9)	0.027(0)
O4	4c	−0.2008(6)	0	−0.3022(4)	0.025(1)
O5	4d	0	−0.2696(8)	−0.1963(7)	0.022(2)
**Bond length (Å)**				**Bond angle(°)**	
Pb-O1: 2.708(6)	Ti-O1: 1.901(8)		Hg1-O1: 2.853(4)	∠Ti-O1-Ti: 152.07(9)	
Pb-O2: 2.701(6)	Ti-O2: 2.005(6)		Hg1-O3: 2.580(1)	∠Ti-O2-Ti: 157.85(3)	
Pb-O3: 2.907(2)	Ti-O3: 1.853(2)		Hg1-O5: 2.331(4)	∠Ti-O3-Ti: 160.25(5)	
Pb-O4: 2.801(7)	Ti-O4: 2.098(0)		Hg2-O1: 2.220(1)	∠Ti-O4-Ti: 145.80(6)	
Pb-O5: 2.569(6)	Ti-O5: 2.102(0)		Hg2-O2: 2.814(7)	∠Ti-O5-Ti: 155.11(8)	
			Hg2-O4: 2.179(1)		
			Hg3-O2: 2.360(5)		
			Hg3-O3: 2.547(8)		

Space group: *Imm*2 (No. 44) *a* = 7.7568(9) Å, *b* = 7.7040(1) Å, *c* = 7.7019(8) Å; *α* = *β* = *γ* = 90°; *V* = 460.26 Å^3^; Z = 2; *R*_p_ = 4.08%, *R*_wp_ = 3.39%.

### First principles calculations

**Fig. 6:**
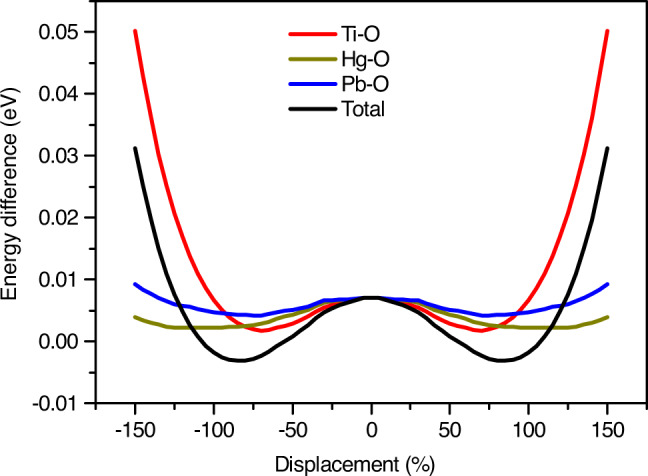
First principles calculation results of the energy changes with respect to the different moving mode along one of the soft mode on Γ point. The red, yellow, blue lines correspond to Ti-O displacements only, Hg-O displacements only and Pb-O displacements only. The black line corresponds to all atoms displacements. The double well structure of the graph indicates the spontaneous structure phase transition.

In order to get a deeper insight into the ferroelectricity of PHTO at low temperature, first principles calculations have been performed. Supplementary Fig. S9 shows the phonon spectrum for the cubic structure of PHTO: three imaginary frequency modes with irreducible representation *T*_*u*_ degenerate at *Γ* point. By moving atoms along these soft phonon modes, the non-centrosymmetric crystal structure with space group *Imm*2 can be obtained. Free energy per primitive cell versus moving amplitude of atoms along one of the soft modes is plotted in Fig. 6. The energy well suggests a spontaneous structure phase transition with the biggest contribution coming from the Ti-O mode. The imaginary modes at *H* point only consist of the Ti-O phonon modes, which are consist with  the experimental conclusion. In addition, the 6*s*^2^ lone pair electrons of Pb^2+^ also contribute to the site instability according to the theory analysis. However, the contribution from the lone pair mechanism does not play a dominant role from the energy perspective (see Fig. 6). Using Berry phase method, the polarization of ferroelectric structure and Born effective charge have been calculated. The calculated polarization value is about 16.87 μC cm^−2^, which can  also be obtained by multiplying the Born effective charge tensor with the corresponding atom displacements. This result is roughly in agreement with that ionic spontaneous polarization calculation result of 13.65 μC cm^−2^ based on neutron diffraction measurements. Both values are larger than the experimental result. This is because the calculated value is based on single crystal data, while the experimental measured value is from a polycrystalline sample of randomly distributed grains. For example, in ceramic samples of BiFeO_3_, much lower values of about 8.9 μC cm^−2^ of polarization have been measured at room temperature while in single crystals the polarization is up to 60 μC cm^−2^ along [012] axis and 100 μC cm^−2^ along [001] axis^48,49^. Another reason for the low experimental value of polarization may be due to the grain boundary effect and/or leaking current issue preventing the application of maximum electric field in the experiment^48,50^. Supplementary Fig. S10 shows the SEM image of the polycrystalline sample of PHTO. It can be seen that the grain size distributes around several microns to nearly 20 microns and generates a large amount of grain boundaries. Those grains are disorderly distributed, which will severely affect the polarization.

The A-site-ordered perovskite PbHg_3_Ti_4_O_12_ was synthesized at high pressure and high temperature. PbHg_3_Ti_4_O_12_ is a nonmagnetic insulator with a direct energy gap of ~2.12 eV and relative dielectric constant larger than 220. PbHg_3_Ti_4_O_12_ undergoes a transition from a high temperature centrosymmetric, cubic and paraelectric phase to a low temperature non-centrosymmetric, orthorhombic and ferroelectric phase with a record high Curie temperature at 250 K for an A-site-ordered quadruple perovskite. The ferroelectric distortion is dominated by Ti-O phonon mode anomaly. Because of the large average size of the A-site cation ~ 1.1 Å in PHTO, the coordination of Hg^2+^ is not square planar as in typical A-site-ordered quadruple perovskites, but closer to 8. Therefore PHTO can be considered to be structurally linking ABO_3_ simple and AA′_3_B_4_O_12_ quadruple perovskites. The established A-site-ordered perovskites AHg_3_B_4_O_12_ may provide a potential pathway to find a class of ferroelectric materials of high Curie temperature. The work provides one of unique examples that high pressure synthesis can lead to the compounds with unique properties that are otherwise hard to get access at ambient. Moreover perovskites are the most abundant minerals in the broad lower Mantle of the Earth where high pressure is prevailing. Hence it might shed lights to further understand the coordination chemistry of perovskites like compounds at high pressures.

## Methods

### Sample fabrication

The polycrystalline sample of PHTO was synthesized by a solid state reaction under high pressure of 6 GPa and high temperature of 1273 K. The starting materials HgO (Aldrich, 99.0% pure), PbO (Alfa, 99.995% pure) and TiO_2_ (Alfa, 99.995% pure) were mixed homogenously in a molar ratio 1:3:4 and pressed into a pellet with diameter of 6 mm in an argon gas protected glove box with oxygen and H_2_O level of less than 1 ppm. The pre-pressed pellet was sealed in a gold capsule. High pressure experiments were performed with the cubic anvil type high pressure apparatus^51,52^. After the pressure was gradually increased to 6 GPa, the sample was heated to 1273 K and maintained for 30 min. Then the temperature was quenched to ambient before the release of pressure. In this process, yellow polycrystalline PHTO was obtained.

### Structure characterization

The crystal structure was characterized by neutron powder diffraction (NPD) with the BT-1 high-resolution neutron powder diffractometer with a Ge (311) monochromator at the Center for Neutron Research (NCNR) of the National Institute of Standard & Technology (NIST). The neutron wavelength was 2.0774 Å. The intensities were measured with steps of 0.05° in the 2*θ* range of 10°–160°. The sample was also measured by synchrotron x-ray diffraction (SXRD) at the 11-BM-B at the Advanced Photon Source (APS) in Argonne National Laboratory. The X-ray wavelength was 0.412726 Å. Diffraction data were collected in the angle (2*θ*) range from 0.5° to 50° with steps of 0.002°. The obtained NPD and SXRD data were analyzed by the Rietveld method with the GSAS program^53^.

### Valences determination

The valence states of the Ti, Pb and Hg ions were determined by x-ray absorption spectroscopy (XAS). The soft XAS at the Ti-*L*_2,3_ edge was measured with total the electron yield mode at the Dragon beamline while the hard XAS at the Pb-*L*_3_ and Hg-*L*_3_ edge were measured with transmission geometry at the BL07A beamline at National Synchrotron Radiation Research Center (NSRRC) of Taiwan. SrTiO_3_, PbTiO_3_, PbNiO_3_ and HgO were also measured at the respective Ti-*L*, Pb-*L* and Hg-*L* edges as reference materials.

### Permittivity and ferroelectricity characterization

The permittivity was measured with different frequency by an Agilent-4980A LCR meter on a solid pellet with 4.0 mm in diameter and 230 μm in thickness. The ferroelectric hysteresis loops were measured at 20 Hz with a Radiant Precision Premier-II Ferroelectric Test System at different temperatures based on the proposed positive-up negative-down (PUND) method. Detailed experimental descriptions can be found in Ref. ^20^.

## Supplementary information

Supplementary Information

## Data Availability

The data that support the findings of this study are available from the corresponding authors upon reasonable request.

## Supplementary information

**Supplementary information** The online version contains supplementary material available at 10.1038/s41467-020-20833-6.
