# Supplementary material for: A combinatory ferroelectric compound bridging simple ABO3 and A-site-ordered quadruple perovskite
Source: Nat Commun. 2021 Feb 2;12:747. doi: 10.1038/s41467-020-20833-6 (PMC7854592; doi:10.1038/s41467-020-20833-6)
Supplement: Supplementary file 1 — Supplementary Information [file 41467_2020_20833_MOESM1_ESM.pdf]

The Supporting Materials

A Combinatory Ferroelectric Compound Bridging Simple ABO_3 and A-site-Ordered Quadruple Perovskite

Jianfa Zhao[1,2], Jiacheng Gao[1], Wenmin Li[1], Yuting Qian[1], Xudong Shen[1,2], Xiao Wang[3], Xi Shen[1], Zhiwei Hu[3], Cheng Dong[4], Qingzhen Huang[5], Lipeng Cao[1], Zhi Li[6], Jun Zhang[1], Chongwen Ren[1], Lei Duan[1], Qingqing Liu[1], Richeng Yu[1], Yang Ren[7], Shih-Chang Weng[8], Hong-Ji Lin[8], Chien-Te Chen[8], Liu-Hao Tjeng[3], Youwen Long[1,2], Zheng Deng[1], Jinlong Zhu[9], Xiancheng Wang[1], Hongming Weng[1*], Runze Yu[1*], Martha Greenblatt[10], and Changqing Jin[1,2*]

Supplementary Methods

The magnetic susceptibility was measured by using a vibrating sample magnetometer (VSM) of a Quantum Design MPMS system. The electrical transport properties are studied using a Quantum Design Physical Properties Measurement System (PPMS) by standard four-probe method. The thermal stability of the sample was measured by thermogravimetry analysis from room temperature to 1473 K at a heating rate of 10 K min^{-1}, using a LABSYS EVO TGA system. Diffuse-reflectance spectra were measured at room temperature using a UV-Vis-NIR spectrophotometer. The morphology and particle size were investigated by Scanning Electron Microscope (SEM) using a field emission scanning microscope (model Hitachi S-4800)

First principles calculation was performed by using the Vienna ab initio simulation package (VASP)[1, 2], which implements the density functional theory with generalized gradient approximation (GGA)[3] of Perdew-Burke-Ernzerhof (PBE) type to exchange-correlation functional. Electronic band structure was calculate with plane wave cutoff of 700 eV and a 7×7×7 Monkhorst-Pack k-point mesh. The phonon band structure were calculated by using the density functional perturbation theory (DFPF) performed with the VASP and PHONOPY package[4]. A 2×2×2 supercell of 160 atoms was used here. We also checked the phonon band result using the ALAMODE package[5], which gave a same result. All these calculations were done after a careful optimization with the given symmetry until the residual Hellmann-Feynman forces became smaller than 10^{-4} eV $Å^{-1}$.

Table S1 | Refined structure parameters of PHTO based on SXRD data collected at 300 K.

	SXRD
a(Å)	7.7234(9)
Z	2
Formula weight	1192.56
Cacl. Density (g/cm^3)	8.5958(9)
V($Å^3$)	460.72(5)
O_y	0.7069(2)
O_z	0.2181(0)
U_{iso}(Pb)($Å^2$)	0.011(4)
U_{iso}(Hg)($Å^2$)	0.009(7)
U_{iso}(Ti)($Å^2$)	0.004(0)
U_{iso}(O)($Å^2$)	0.003(2)
Pb-O(×12)(Å)	2.821(6)
Hg-O(×4)(Å)	2.322(1)
Hg-O(×4)(Å)	2.700(8)
Ti-O(×6)(Å)	1.974(7)
∠Ti-O-Ti (°)	155.80(1)
BVS(Pb)	1.76
BVS(Hg)	2.11
BVS(Ti)	3.90
R_{wp}(%)	8.47
R_p(%)	6.79

[a] The BVS values (V_i) were calculated using the formula $V_i = \Sigma_j S_{ij}$, and $S_{ij} = \exp[(r_0 - r_{ij})/0.37)]$. In PHTO, r_0 =2.112 for Pb, 1.972 for Hg and 1.815 for Ti. For the A-site Pb, 12-coordinated oxygen atoms were used. For the A′-site Hg, 8-coordinated oxygen atoms were used. For the B-site Ti, 6-coordinated oxygen atoms were used. [b]Space group: *I*m-3; Atomic sites: Pb 2*a* (0, 0, 0), Hg 6*b* (0, 0.5, 0.5), Ti 8*c* (0.25, 0.25, 0.25), O 24*g* (*0*, *y*, z).

Table S2 | Refined structure parameters of PHTO based on SXRD data collected at 90 K.

Crystallographic data for $PbHg_3Ti_4O_{12}$ based on SXRD at 90 K					
Atom	Wyck	x	y	z	U_{iso} (Å^2)
Pb	2a	0.5	0.5	0.5	0.008(9)
Hg1	2b	0.5	0	0.5	0.007(1)
Hg2	2a	0.5	0.5	0	0.005(1)
Hg3	2b	0.5	0	0	0.005(4)
Ti	8e	0.2319(7)	0.2547(5)	0.2295(1)	0.016(2)
O1	4c	0.2125(1)	0	0.2707(9)	0.002(4)
O2	8e	0.2872(7)	0.1966(5)	-0.0179(9)	0.010(4)
O3	4d	0	0.3061(7)	0.2208(8)	0.005(1)
O4	4c	-0.1982(8)	0	-0.2835(1)	0.005(0)
O5	4d	0	-0.2942(1)	-0.2414(7)	0.001(7)

**Bond length (Å)**			**Bond angle(°)**
Pb-O1: 2.657(1)	Ti-O1: 1.993(9)	Hg1-O1: 2.842(1)	∠Ti-O1-Ti : 159.67(0)
Pb-O2: 2.696(1)	Ti-O2: 2.004(2)	Hg1-O3: 2.263(5)	∠Ti-O2-Ti : 150.81(7)
Pb-O3: 2.908(2)	Ti-O3: 1.841(7)	Hg1-O5: 2.443(7)	∠Ti-O3-Ti : 154.80(5)
Pb-O4: 2.669(8)	Ti-O4: 1.967(7)	Hg2-O1: 2.411(4)	∠Ti-O4-Ti : 147.56(0)
Pb-O5: 2.931(9)	Ti-O5: 2.122(5)	Hg2-O4: 2.267(1)	∠Ti-O5-Ti : 156.15(6)
		Hg3-O2: 2.243(0)	
		Hg3-O3: 2.617(4)	
		Hg3-O5: 2.545(1)	

Space group: *Imm*2（No. 44）a = 7.7483(9) Å, b = 7.7042(4) Å, c = 7.7014(2) Å; $\alpha = \beta = \gamma$ = 90°; V = 459.740 Å^3; Z = 2; R_p = 5.34%, R_{wp} = 6.95%.

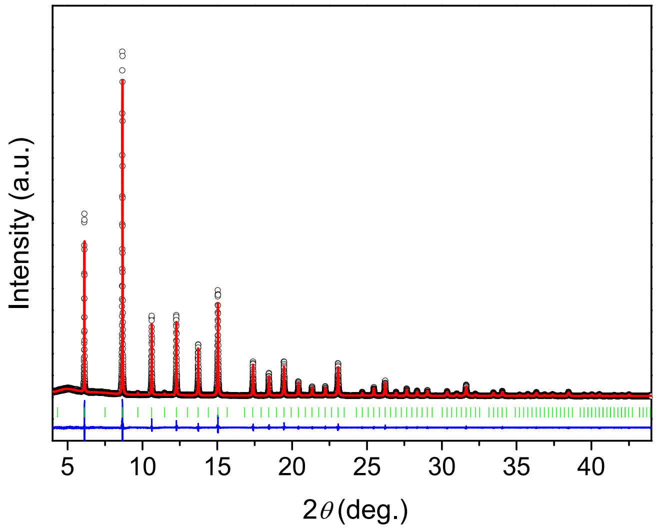

Fig. S1 Rietveld refinements of SXRD patterns for at 300 K PHTO. Observed (crosses), calculated (red), difference (blue) and Bragg reflections (green) are shown in the figure, respectively. The ticks indicate the allowed Bragg reflections with space group *Im*-3.

a

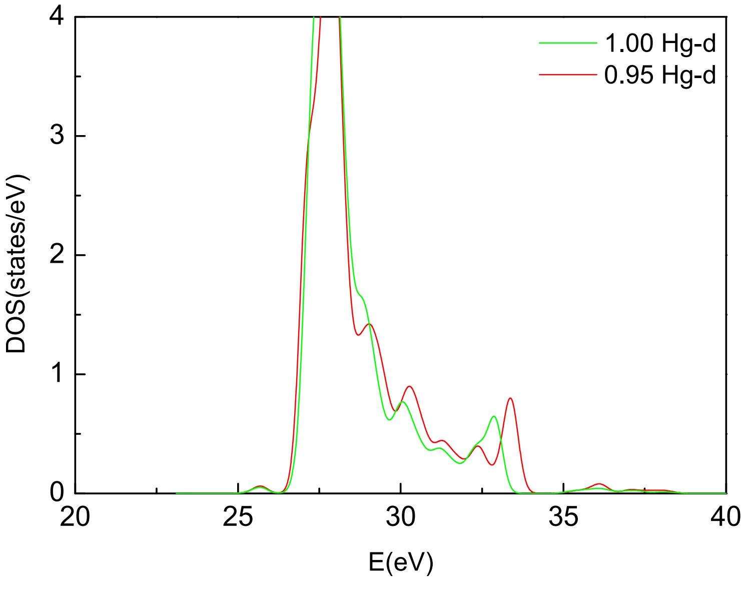

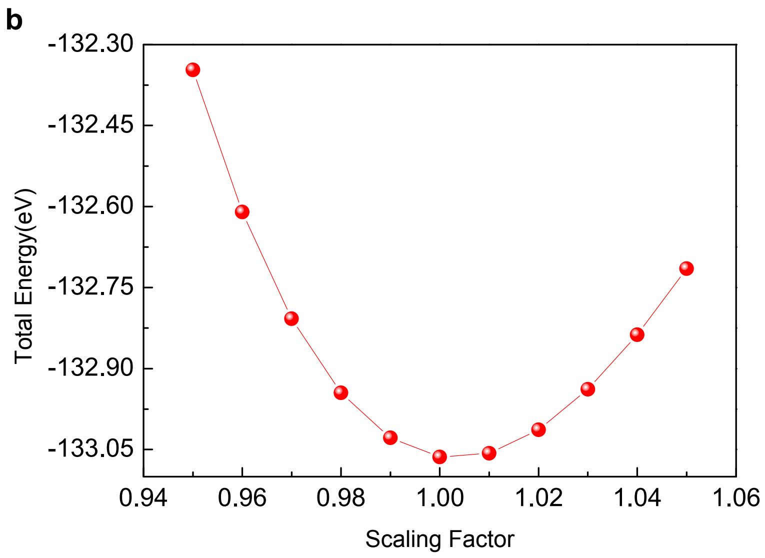

Fig. S2 **a**, The density of states projected on the *d* orbitals of a single Hg atom. Systems with smaller Hg-O scaling factor have higher d^{10} energy level. The energy is aligned with respect to the Ti 3*p* states so that the energy positions can be directly compared. **b**, The total energy of PHTO system as a function of the Hg-O bond length. The shorter bond of Hg-O is scaling factor times the original bond length.

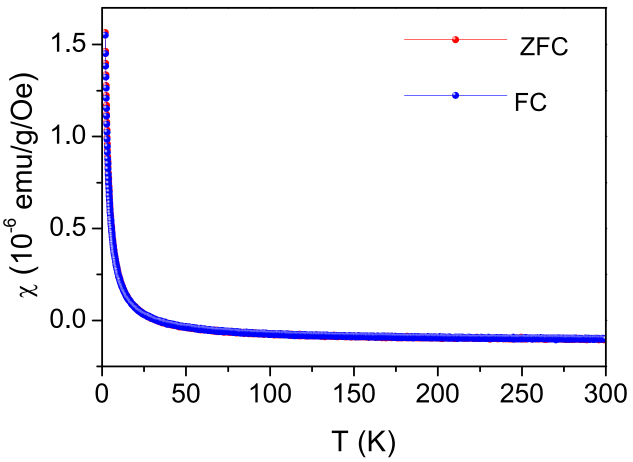

Fig. S3 Temperature dependent magnetic susceptibility χ for PHTO with external magnetic field of 0.1 T.

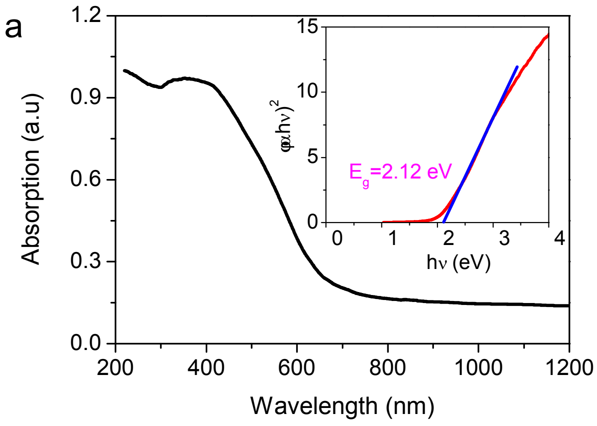

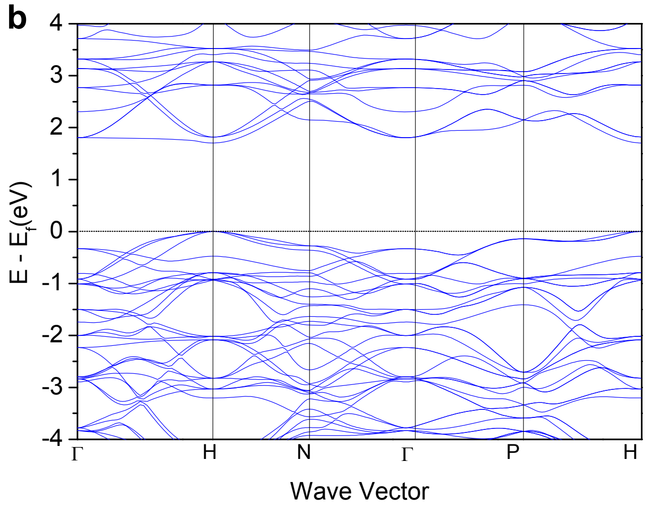

Fig. S4 a, UV-Vis-NIR diffuse reflectance spectrum of PHTO measured at room temperature. **b**, First-principle calculation of PHTO band structures.

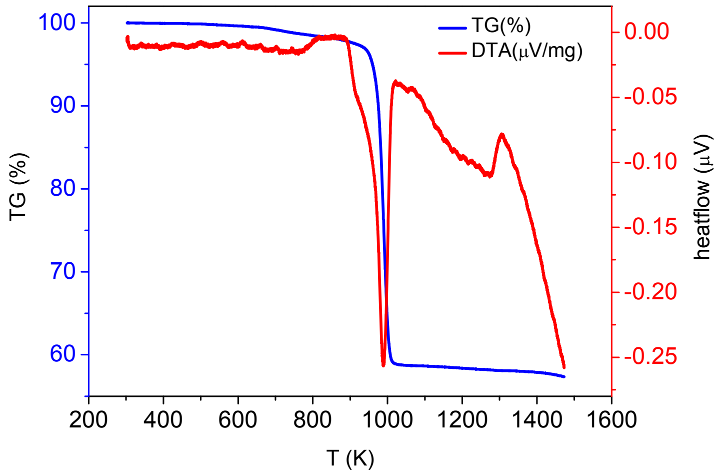

Fig. S5 Thermogravimetry analysis of PHTO. The TGA measurement results show that the sample decomposed at about 973 K, losing about 40% of its mass. The residual products were TiO_2 and PbO, so the decomposition reaction was $PbHg_3Ti_4O_{12} \rightarrow PbO+3HgO+4TiO_2$. The observed weight loss agrees well with the HgO, which evaporates into the air at high temperature.

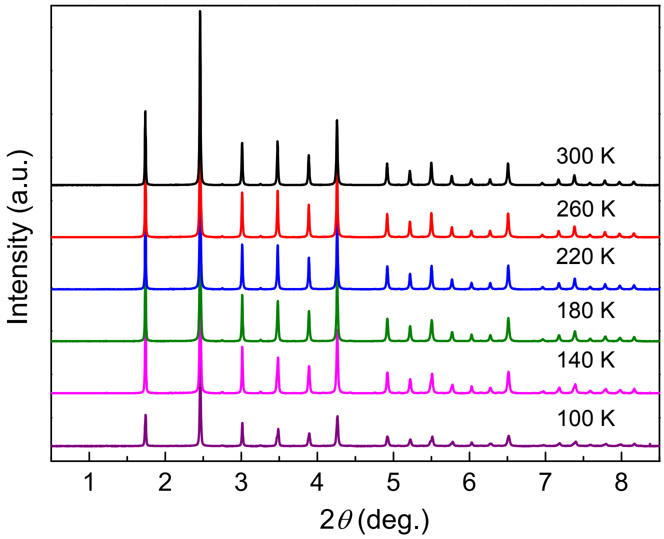

Fig. S6 Temperature dependence of SXRD patterns of PHTO obtained from 300 K to 100 K.

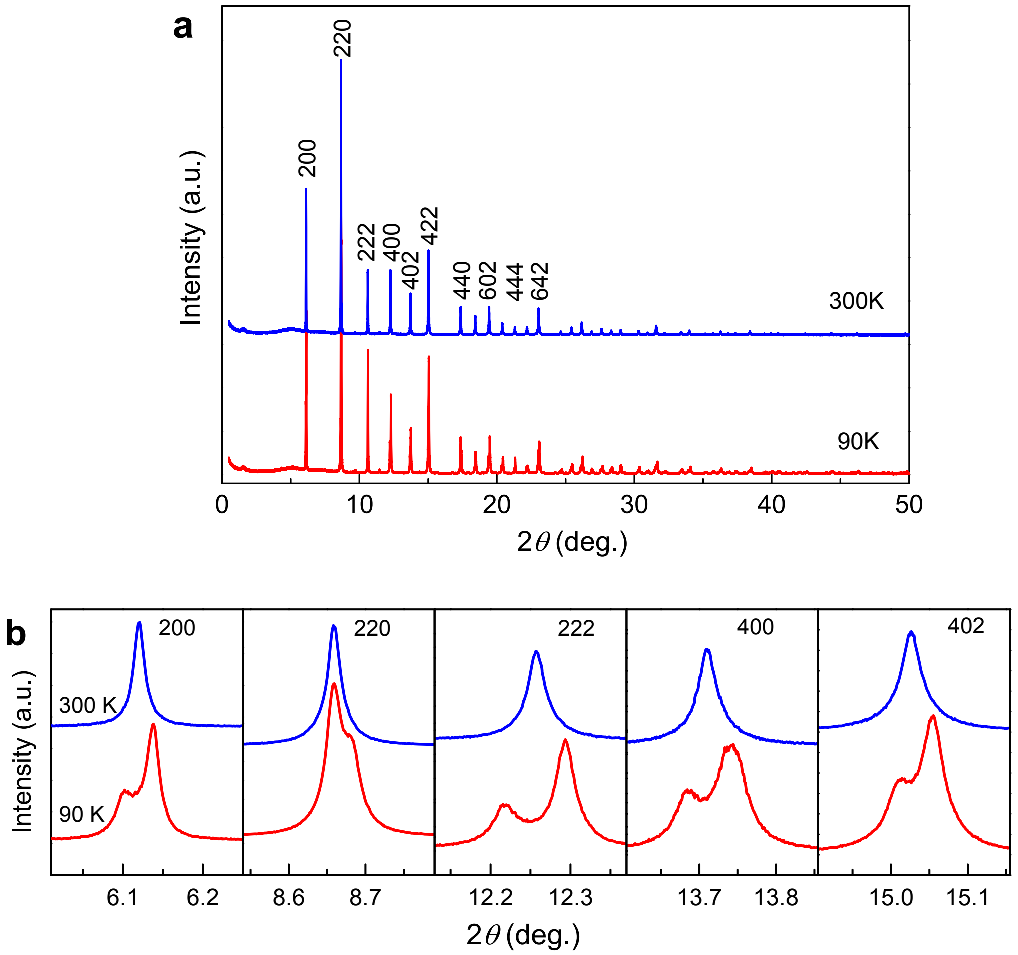

Fig. S7 **a**, SXRD patterns of PHTO obtained at 300 K and 90 K. **b**, Some characteristic diffraction peaks collected at 300 K and 90 K.

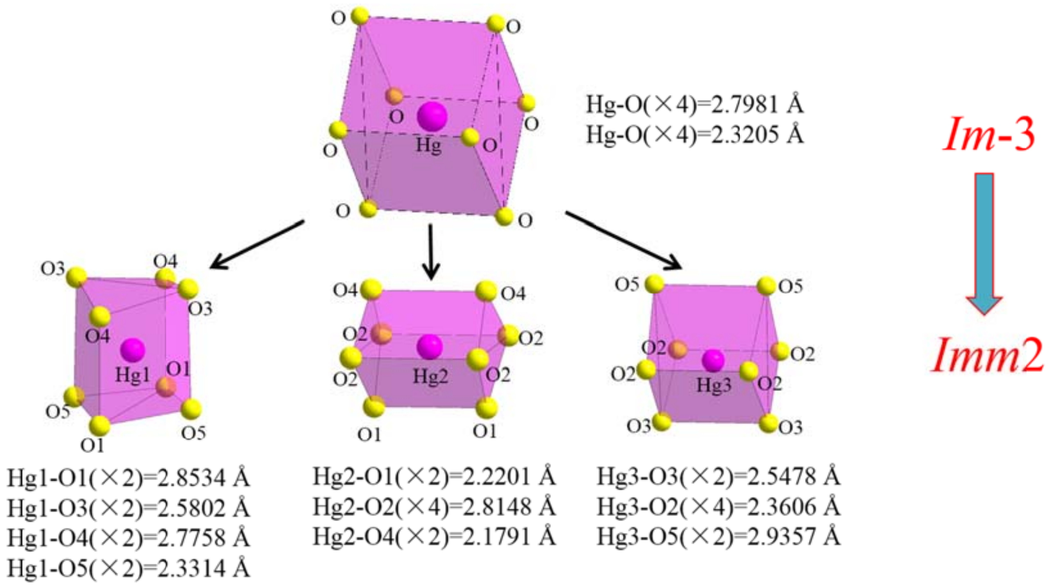

Fig. S8 Schematic illustration for the changing of HgO_8 hexahedron before and after phase transition

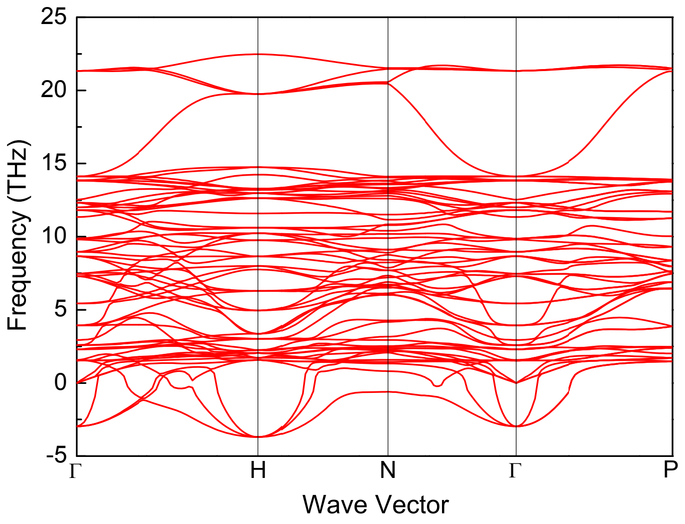

Fig. S9 The phonon band calculated by using the DFPT method.

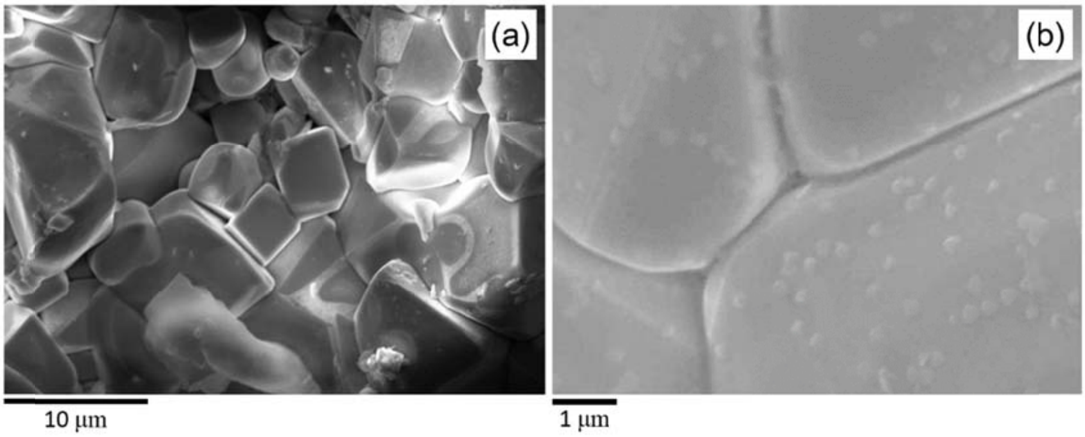

Fig. S10 SEM micrographs of **a**, the fracture surface and **b**, grain boundaries for polycrystalline sample of PHTO.

REFERENCES

1 Kresse, G. & Furthmuller, J. Efficient iterative schemes for ab initio total-energy calculations using a plane-wave basis set. *Phys. Rev. B* **54**, 11169-11186 (1996).

2 Kresse, G. & Joubert, D. From ultrasoft pseudopotentials to the projector augmented-wave method. *Phys. Rev. B* **59**, 1758-1775 (1999).

3 Perdew, J. P., Burke, K. & Ernzerhof, M. Generalized gradient approximation made simple. *Phys. Rev. Lett.* **77**, 3865-3868 (1996).

4 Togo, A., Oba, F. & Tanaka, I. First-principles calculations of the ferroelastic transition between rutile-type and $CaCl_2$-type SiO_2 at high pressures. *Phys. Rev. B* **78**, 134106 (2008).

5 Tadano, T., Gohda, Y. & Tsuneyuki, S. Anharmonic force constants extracted from first-principles molecular dynamics: applications to heat transfer simulations. *J. Phys.: Condens. Matter* **26**, 225402 (2014).
